# OMGene: mutual improvement of gene models through optimisation of evolutionary conservation

**DOI:** 10.1186/s12864-018-4704-z

**Published:** 2018-04-27

**Authors:** Michael P. Dunne, Steven Kelly

**Affiliations:** Department of Plant Sciences, University of Oxford, South Parks Road, Oxford, OX1 3RB UK

**Keywords:** Genome annotation, Annotation errors, Orthogroups, Orthology, Gene model

## Abstract

**Background:**

The accurate determination of the genomic coordinates for a given gene – its *gene model –* is of vital importance to the utility of its annotation, and the accuracy of bioinformatic analyses derived from it. Currently-available methods of computational gene prediction, while on the whole successful, frequently disagree on the model for a given predicted gene, with some or all of the variant gene models often failing to match the biologically observed structure. Many prediction methods can be bolstered by using experimental data such as RNA-seq. However, these resources are not always available, and rarely give a comprehensive portrait of an organism’s transcriptome due to temporal and tissue-specific expression profiles.

**Results:**

Orthology between genes provides evolutionary evidence to guide the construction of gene models. OMGene (Optimise My Gene) aims to improve gene model accuracy in the absence of experimental data by optimising the consistency of multiple sequence alignments of orthologous genes from multiple species. Using RNA-seq data sets from plants, mammals, and fungi, considering intron/exon junction representation and exon coverage, and assessing the intra-orthogroup consistency of subcellular localisation predictions, we demonstrate the utility of OMGene for improving gene models in annotated genomes.

**Conclusions:**

We show that significant improvements in the accuracy of gene model annotations can be made, both in established and in *de novo* annotated genomes, by leveraging information from multiple species.

## Background

The utility of any given genome is dependent on the comprehensiveness and accuracy of its gene model annotations. Inaccuracies in the annotated locations and structures of protein coding genes can lead to myriad downstream errors. These include misinformed conclusions about the biological properties of an organism, as well as errors in transcript quantification, phylogenetic tree inference, protein localisation, and protein structure predictions. It is therefore vital to downstream analysis, both computational and experimental, to ensure that gene annotations are as accurate as possible.

The absolute quantity of publicly available genomic data has grown exponentially over the past two decades, as has the number of taxa represented [1–3], owing to the consistently decreasing costs of acquiring whole genome sequences [4, 5]. Accordingly, the feasibility of manual gene model annotation has diminished progressively, with a corresponding increase in reliance on computational gene prediction software. As such there are numerous tools available for the *de novo* and data-assisted prediction of genes [6]. These tools typically rely on genetic signatures such as GC content, codon bias, feature length distributions, and various conserved DNA sequence motifs. Though many of these tools are highly proficient at gene prediction, mistakes are common. Gene prediction tools often disagree on the quantity of genes that they predict [7–9]. Furthermore, even when gene predictors agree on the location of a gene, the predicted intron-exon structure for that gene can vary considerably between the different methods [10]. Common errors include erroneous exon/intron retention/omission, inaccurate exon/intron boundaries, frame errors, misplaced start codons, and fragmentation/fusion of gene models.

When available, the use of extrinsic empirical data, most notably RNA-seq, is the most reliable currently available method for procuring gene models. For example, single contiguous RNA-seq reads obtained from mRNA sequencing can be split across multiple loci when mapped to the genome, providing evidence for the locations of splice junctions. Unfortunately, empirical data is generally not available for all genes in a given species: many genes are expressed in a cell-type or cell-cycle specific manner and for organisms with many disparate tissue types it can be difficult to obtain RNA-seq data that cover the full breadth of the transcriptome [11, 12]. In addition, not all gene sequences are amenable to reliable and accurate alignment, in particular identical duplicate genes and genes that contain repetitive regions found in multiple other genes [13]. Furthermore library preparation protocols and other statistical factors can make reliable gene model inferences difficult [14–16]. Finally, there are some aspects of gene models that are simply not revealed by RNA-seq analysis: for example the presence of 5’UTR sequences or internal methionine residues mean that there can often be multiple plausible start codons locations for a given open reading frame (ORF), and so start codon location cannot be inferred from RNA-seq data.

Feature locations (splice sites, exons, transcription start sites) have been shown to be highly conserved across evolutionary timescales, often more so than the constituent amino acid sequences they encapsulate [17, 18], despite alternative splicing being a driver of divergence [19]. Given various gene model predictions, if multiple highly similar (in sequence and structure) gene models exist for a gene across multiple taxa, they are more likely to be biologically correct than disparate alternatives. Several de novo gene prediction algorithms have utilised this concept to constrain gene searches by predicting genes in multiple genomes simultaneously: notably SLAM [20], SGP-1 [21], TWINSCAN/N-SCAN/CONTRAST [22, 23], and the most recent version of Augustus [24]. However, no tool currently exists that can systematically and automatically improve the annotations of already predicted genes by leveraging annotated gene models from other species.

By considering *orthogroups* of related genes, one can optimise the similarity of gene models across species by seeking conserved structure across the various taxa. In the absence of extrinsic data, it is parsimonious to choose gene models that maximise intra-orthogroup amino acid alignment agreement. OMGene (Optimise My Gene) aims to improve genome annotations by optimising the agreement between gene models for orthologous genes in multiple species. It is designed to function without the need for additional empirical data, utilising only the local genome sequences for the genes in question, and works on existing predicted gene models. A standalone implementation of the algorithm is available under the GPLv3 licence at https://github.com/mpdunne/omgene. The algorithm is available as a python script, instructions for which, along with example data sets, are included in the git repository.

### Implementation

#### Algorithm description

The input for OMGene is a set of gene model files in general transfer format (GTF) and a set of corresponding FASTA genome files. GTF is a variant of GFF3 with a more standardised structure. As many public gene datasets use GFF3 format, a tool for extracting well-formed gene entries from GFF3 files is included in the GitHub repository. Each GTF file should contain coordinates for one gene only. The FASTA file can contain one or multiple sequences but must contain the nucleotide sequence referenced by the GTF file. The set of GTF files and their corresponding genome files are inputted to OMGene using a tab-delimited CSV file: each line in the input should contain first the file path to a gene model’s GTF file, followed by the path to the genome for that species. If the GTF contains multiple transcript variants then these are considered together as variants of a single gene. Further information regarding input formats can be found in the OMGene GitHub repository.

For each inputted gene, the algorithm defines its *gene region* to be the region spanning the first and last base of any of its corresponding gene models, with a user-selected number of buffer bases either side (default value is 600 bp). The initial step of OMGene is to cross-align the amino acid sequences from each gene with the gene regions of the other genes using Exonerate [25]. The rationale behind this step is to find exonic regions that are present in one or more gene models but absent from one or more annotated gene model. This is performed three times: first by cross-aligning the input protein sequences against all gene regions, second by cross-aligning the protein sequences that have been found in the first step against all gene regions, and finally by cross-aligning all individual exon sequences from the first step. This three-step process mitigates against lack of detection due to gene model errors in one or more of the input genes. This, together with the exons from the original gene sequences, comprises a set of potential gene parts, which may overlap and which may be incompatible in reading frame. Compatible combinations of gene parts (i.e. without frame-shift errors) are strung together to form a putative gene model. Many such putative gene models may exist: the set of putative gene models with the highest alignment score (see Alignment score calculation below) is carried forward to the next step. If multiple alternatives have the same alignment score, the choice is made randomly.

The set of putative gene models from the previous step are aligned, and the set of putative exons from all genes is divided into *adjacency groups*: sets of exons that overlap each other in the alignment (see below). Exons are added in sequentially in these adjacency groups, and at each stage a valid gene model is sought on the left hand side of the gene (i.e. starting at the start codon and seeking to adjoin exons in valid donor-acceptor pairs). Multiple options for each gene are produced at each new junction, by recursively seeking out, or “wiggling” splice junctions (or start codons) in each frame either side of the existing exons start and end points. This produces a set of junction options for each pair of exon ends. A multipartite choice function is then used to choose the optimal set of exons across all secies, as described below. In the event that a particular exon is very small (< 40 bp), or does not yield any valid junction sites, both that exon and the one before it are probed for removal, and the variant with the removed exon is compared against the other partial gene models in the evaluation step. Once this recursive step ceases to produce new gene modes, the gene model set with the highest alignment score is declared the winner, and the next putative exon from the next adjacency group is added. If multiple alternatives have the same alignment score, the choice is made randomly. This is repeated until there are no further exons to add.

To ensure that the optimisation process did not overlook potentially better variants in the user-supplied gene models, the process above is repeated. This time, instead of varying exons start and end sites, the set of newly created junctions are compared against the original junctions, aiming to find the optimal combination of new and old junctions.

The final step involves filtering the changes based on a selection of categories that have been observed to over-fix gene models. Firstly, we require the alignment score *α* of a 10 amino acid region each side of the change to have either remained the same or improved. This is a basic requirement which should be met in most cases due to the way in which sequence variants are chosen. Secondly, changes that have opened gaps in the alignment in three or more of the sequences are not allowed: this is a common occurrence due to sequences proximal to exon termini that by chance feature valid splice junction sequences that are in frame with the adjacent exons and are evolutionarily conserved. These tend not to be correct. Thirdly, very small changes are forbidden: changes that have resulted in two or fewer amino acids being changed in a gapless region of the alignment, such that the new alignment is also gapless, are ignored. Similar changes to larger regions require an *α* (see Alignment score below) increase of 4 or more. This is to avoid changes that reflect multiple choices of donor-acceptor pairs for essentially identical sequences. Finally, the alignment in the region of the change must be of reasonable quality: for unchanged 5 amino acid regions either side of the region under consideration, the adjusted alignment score $\overline{\alpha}$ must be 3 or higher (or all gaps) for some subset of three sequences containing the sequence of interest. Similarly the resulting score for the changed region must also be higher than 3 or all gaps. Exon boundaries that do not pass the filters are discarded and the genes are reconstructed a final time, allowing only the surviving boundaries and those that were present in the original gene. The resultant genes are outputted in GTF, amino acid FASTA and CDS FASTA format.

### Data sources

For algorithm development and evaluation, three species sets were selected (Table 1): a set of five fungal genomes, a set of five plant genomes, and a set of five mammal genomes. Each species set contained at least one well-annotated model organism. Orthogroups were inferred using OrthoFinder [26]. For the plant data set, where multiple transcript variants were available, the primary transcript was used as listed in Phytozome [27]. For the mammal data set, no primary transcripts were marked, and so for each gene the longest available transcript was used. RNA-seq data sources are listed in Table 2. and were downloaded from the Sequence Read Archive [28].

**Table 1:** Species sets used for algorithm validation

	Species Name	Source	Version/Strain	Taxonomy ID	References
*Plant species*	*Arabidopsis thaliana*	JGI^1^	TAIR10	3702	[39, 40]
	*Brassica rapa*	JGI	v1.3	3711	[27]
	*Carica papaya*	JGI	ASGPBv0.4	3649	[39, 41]
	*Capsella rubella*	JGI	v1.0	81,985	[39, 42]
	*Theobroma cacao*	JGI	v1.1	3641	[39, 43]
*Mammalian species*	*Canis lupus familiaris*	NCBI	CanFam3.1/AR105	6915	[44, 45]
	*Homo sapiens*	CCDS^3^	GRCh38.p7/CCDS20	9606	[46]
	*Monodelphis domestica*	NCBI	MonDom5 / AR103	13,616	[45, 47]
	*Mus musculus*	CCDS	GRCm38.p4/CCDS21	10,090	[46]
	*Oryctolagus cuniculus*	NCBI	OryCun2.0/AR102	568,996	[45, 48]
*Fungal species*	*Eremothecium gossypii*	JG	*ATCC10895*	284,811	[49]
	*Debaryomyces hansenii*	JGI	*CBS767*	284,592	[50, 51]
	*Kluyveromyces lactis*	JGI	*CLIB210*	284,590	[50]
	*Saccharomyces cerevisiae*	SGD^4^	*S288C*	559,292	[52]
	*Yarrowia lipolytica*	JGI	*CLIB122*	284,591	[50]

^1^ *Joint Genome Institute;* ^*2*^ *National Centre for Biotechnology Information;* ^*3*^ *Consensus Coding Sequence Project;* ^*4*^ *Saccharomyces Genome Database*

**Table 2:** SRA RNA-seq data sources

	Species	SRA ID	Instrument/details
Plants	*A. thaliana*	SRR3932355	Illumina HiSeq 2500, paired end. Wild type Columbia
	*B. rapa*	SRR2984945	Illumina HiSeq 2000, paired end. ga-deficient dwarf (gad1–2) + GA rep2
	*C. papaya*	SRR3509576	Illumina HiSeq 2500, paired end. SunUp/Sunset cultivar, young hermaphrodite leaf
	*C. rubella*	SRR3993756	Illumina HiSeq 2000, paired end. Leaf sample
	*T. cacao*	SRR3217315	Illumina HiSeq 2000, paired end. Flower/leaf sample
Mammals	*C. lupus*	ERR266386	Illumina Genome Analyzer II, paired end, brain frontal cortex, male
		ERR266355	Illumina Genome Analyzer II, paired end, brain frontal cortex, female
		ERR266382	Illumina Genome Analyzer II, paired end, brain frontal cortex, male
	*H. sapiens*	SRR5938455	Illumina HiSeq 2000, paired end, dorsolateral prefrontal cortex, male
	*M. domestica*	SRR500906	Illumina HiSeq 2000, paired end, brain
		SRR500925	Illumina HiSeq 2000, paired end, brain
	*M. musculus*	SRR5441717	Illumina HiSeq 2000, paired end, brain (striatum)
		SRR6269591	Illumina NovaSeq 6000, paired end, cerebellum
	*O. cuniculus*	ERR266399	Illumina Genome Analyzer II, paired end, brain frontal cortex, female
		SRR400990	Illumina Genome Analyzer II, paired end, brain frontal cortex
		SRR401040	Illumina Genome Analyzer II, paired end, brain frontal cortex
		SRR401041	Illumina Genome Analyzer II, paired end, brain frontal cortex
		SRR401042	Illumina Genome Analyzer II, paired end, brain frontal cortex
Fungi	*K. lactis*	SRR1200528	Illumina Genome Analyzer II, single
	*S. cerevisiae*	SRR539284	Illumina HiSeq 2000, paired end
	*Y. lipolytica*	SRR868669	Illumina HiSeq 2000, single

#### *De novo* gene prediction

*De novo* gene predictions were made using Augustus [24] version 3.2.2. Training was performed using all well-formed gene models from each species, and using the autoAugTrain.pl script included with the software. Augustus was run individually on each genome with the default settings.

### Alignment score

An amino acid alignment can be considered as an ordered sequence $A={\left({C}_n\right)}_{n=1}^{n=l}$ of columns ${C}_n=\left({c}_1^n,\dots, {c}_l^n\right)$. The *column score γ* for a column *C*_*n*_ is defined as the average pairwise Blosum62 score for amino acids in that column:

$$ \gamma \left({C}_n\right)=\frac{\sum_{1\le i<j\le l} Blos\left({c}_i^n,{c}_j^n\right)}{l} $$

The Blosum62 matrix was used as it is the most widely used amino acid substitution matrix. The *alignment score α* for an alignment *A* is constructed column-wise as:

$$ \alpha (A)=\sum \limits_{n=1}^l\gamma \left({C}_n\right) $$

The *adjusted alignment score* $\overline{\alpha}$ is defined as $\overline{\alpha}=\frac{\alpha }{l}$, where *l* is the alignment length.

### Multipartite choice function

**Fig. 1:**
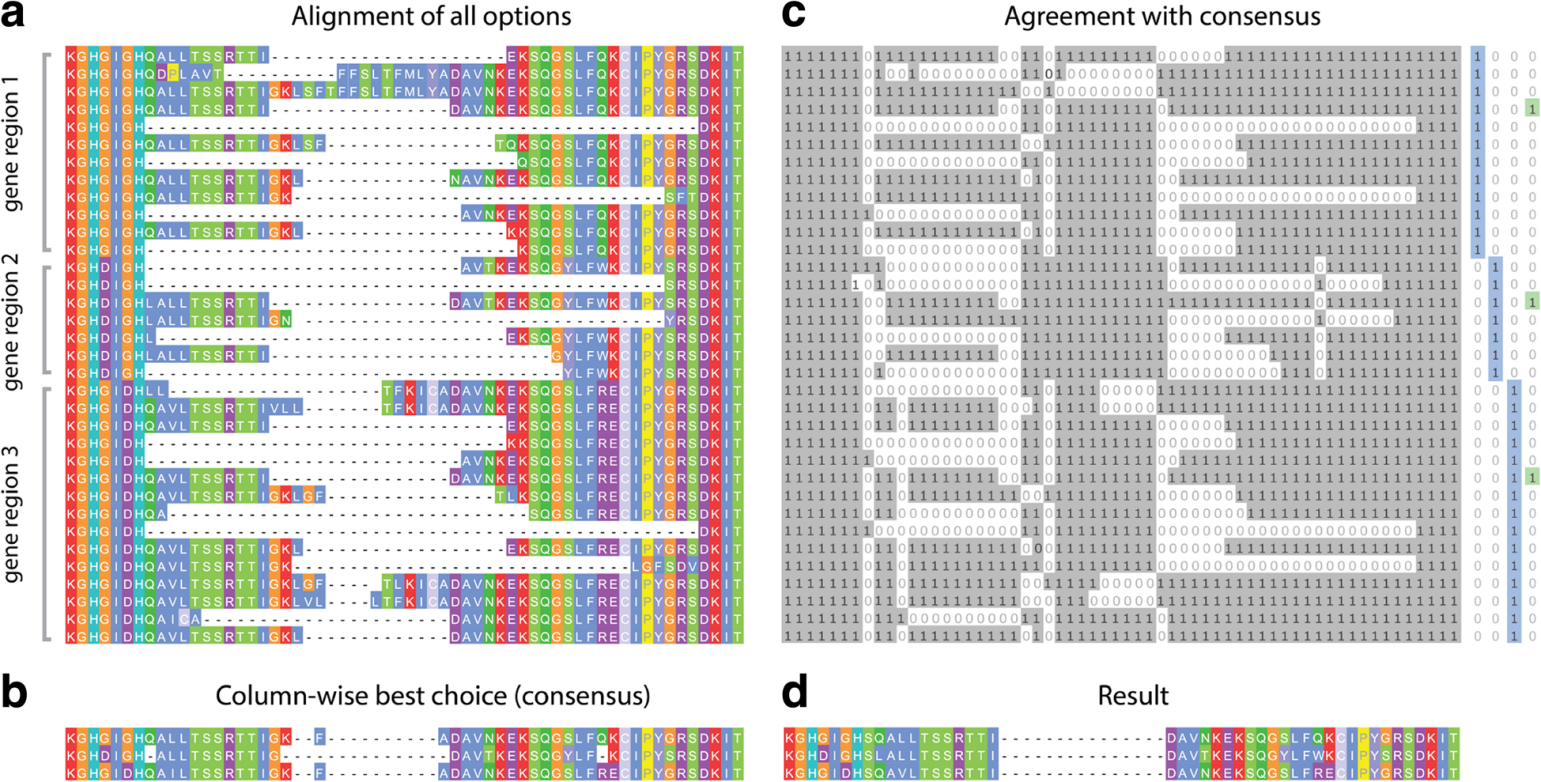
Multipartite Choice Function. The choice function aims to find optimal variants from a set of protein sequences. **a**) Sequences are aligned; **b**) A consensus alignment is produced: on a column-by-column basis the choice of amino acid for each sequence that optimises the alignment score for that column is chosen as a representative; **c**) A binary representation is produced from the original alignment: for each position in alignment, a 1 is assigned if the amino acid matches the consensus, and a 0 is assigned if it does not. This leaves a sequence of vertical binary strings. The aim is to find a single vertical binary string that agrees with (i.e. is a bitwise subset of) as many as possible of these, and that is also compatible with the category constraints. The best such string in this case is shown to the right in green. **d**) The result

The multipartite choice function (Fig. 1) aims, for a set of *k* gene regions and a set of *l*_*k*_ gene model variants for each gene region, to choose an optimal set containing one gene model variant from each gene region such that the alignment score is maximised. This problem is equivalent to finding the heaviest maximal clique in an edge-weighted complete multipartite graph. This function replaces the naïve approach of calculating pairwise alignment scores between all options and choosing an optimal subset, which, while optimal, is computationally infeasible.

To reduce the complexity of the problem, options are chosen by comparison with a reference consensus alignment, produced by taking the most consistent set of amino acids for each column in a global alignment individually (Fig. 1a-b). This column-wise optimisation is fast, and provides a basis for the sequence-wide optimisation. To produce the consensus, The set of ∑*l*_*k*_ options is aligned to the reference (the original alignment) using MAFFT –add [29]. The inconsistent regions are then isolated and re-aligned using the more accurate but more computationally intensive MAFFT L-INS-i. For each column in the alignment, the set of amino acid choices (one for each gene region) that optimises the alignment score for that column is chosen.

For each option *i* a binary string ${H}_i=\left\{{h}_1^i,\dots, {h}_n^i\right\}$ is produced describing for each position in the alignment whether or not that option matches the consensus (Fig. 1c). The chosen subset will be the set of options that globally maximises agreement with the consensus. If the strings {*H*_*i*_}_*i*_ are stacked vertically, such that they can be read as columns ${\left\{{V}_j\right\}}_{j=1}^n$ then the task is equivalent to finding a columnar binary string *V* with one nonzero entry for each gene region such that |*V*_*i*_ : *V* ⊆ *V*_*i*_| is maximised.

Given the set ${A}_0={\left\{{V}_j\right\}}_{j=1}^n$, an optimal subset is deduced by sequential random sampling. Ignoring all-1 strings, an initial *W*_0_ = *V*_*k*_ is chosen at random from *A*_0_. For sets *S*_1_, *S*_2_ and a set of “checkpoints” *R*, the set *S*_1_ is *compatible with S*_2_*with respect to R* = {*R*_*i*_}_*i*_ if the binary intersection *S*_1_ ∩ *S*_2_ ∩ *R*_*i*_ is nonzero for all *i*. Define *A*_*n*_ = {*a* ∩ *W*_*n* − 1_ : *a*, *W*_*n* − 1_ compatible w. r. t *G*}, where *G* is the set of binary strings which are zero for all but one gene region, at each stage choosing *W*_*n*_ at random from *A*_*n*_. The process *A*_0_, *A*_1_, *A*_2_, … eventually converges on a single binary string. This reduction is performed a user-selected number of times, the default being 1000. The result that is a subset of the largest number of *V*_*i*_ is declared the winner. In the event that the result still contains more than one option for each gene region, subsets of options are calculated and their multiple alignment score *α* is calculated, the winner being the subset with the highest *α*. In the event that multiple subsets exhibit the same maximal *α*, a subset is chosen arbitrarily from them.

### Adjacency group calculation

**Fig. 2:**
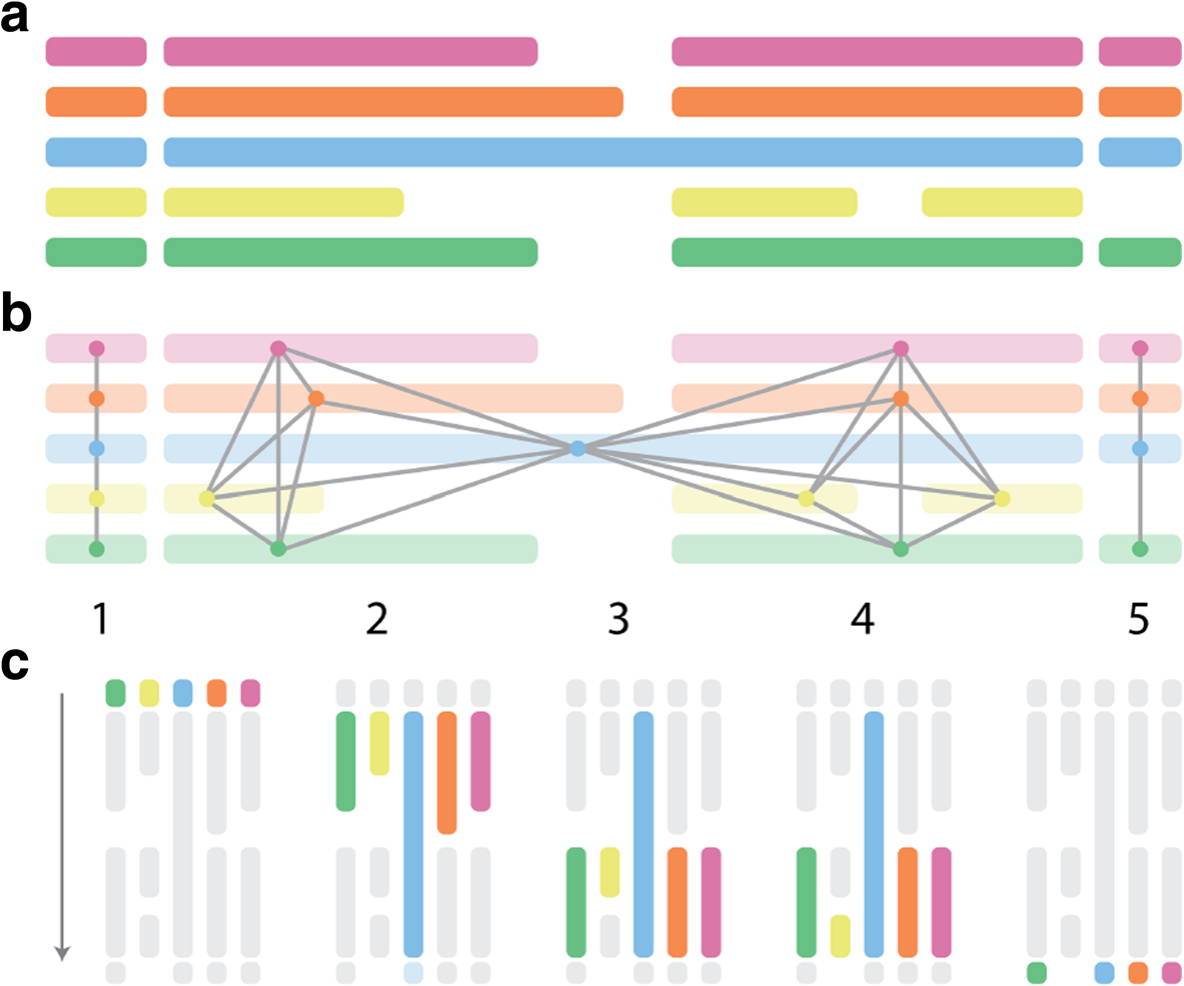
Calculation of adjacency groups. **a**) Amino acid sequences for individual putative exons are strung together and aligned. **b**) A graph is formed with vertices formed by gene parts (or exons), and edges drawn when the overlap between two parts is greater than or equal to one third the length of one of them. **c**) Cliques are extracted and then ordered lexicographically to form the adjacency groups

OMGene builds genes sequentially by iteratively adding in putative exons to multiple genes simultaneously. Care must be taken to ensure the gene parts (which in turn become exons once gene models are constructed) are added in a way conducive to vertical comparison of relevant regions (see Fig. 2). In OMGene, gene parts are considered in sequential *adjacency groups* based on their coordinates in a multiple sequence alignment. Prototype gene models are formed by stringing together amino acid sequences for individual putative exons for each gene region: these are then aligned, and a graph is formed from this alignment. Each putative exon is a node on the graph, and two exons are connected by an edge if and only if one of the exons overlaps the other by a third or more of its length. The adjacency groups are then defined to be cliques in this graph. Cliques are determined using the python implementation of the NetworkX package [30].

### Junction F-score

The *junction F-score* for a gene is a measure of how well the splice junctions observed in mapped RNA-seq data are represented in the gene model. For a gene model *G* and corresponding gene region *R*, define *J*_*G*_ to be the set of individual intron beginning and end coordinates in the gene model, and define *J*_*R*_ to be the set of mapping junction beginning and end coordinates in the mapped RNA-seq data. A minimum of 10 reads is required for a given RNA-seq junction to be counted. We may then define the junction F-score as:

$$ jF\left({J}_G,{J}_R\right)=\frac{2\bullet jP\left({J}_G,{J}_R\right)\bullet jR\left({J}_G,{J}_R\right)}{jR\left({J}_G,{J}_R\right)+ jP\left({J}_G,{J}_R\right)} $$

where

$$ jP\left({J}_G,{J}_R\right)=\frac{\left|{J}_G\cap {J}_R\right|}{\left|{J}_R\right|};\kern1.5em jR\left({J}_G,{J}_R\right)=\frac{\left|{J}_G\cap {J}_R\right|}{\left|{J}_G\right|} $$

The direction of each junction site (start or end of a junction) is taken into account when considering the intersection of the two sets.

### Coverage score

The *coverage score* is a measure of how well RNA-seq data represents a given gene. Given that gene expression levels can vary considerably and irregularly across the length of a transcript [13–16], care must be taken to ensure the expression profile for a gene region is properly interpreted. For example, sample preparation methods can bias coverage towards the centre and 3′ ends of the transcript; furthermore, jagged read profiles and transcription of antisense regions [31] and other intronic ncRNAs can cause expression profiles to be highly non-binary. To mitigate this, a rolling threshold approach is used. For a gene region *R*, and a genomic coordinate *x* ∈ *R*, the expression characteristic *χ* is defined as:

$$ \chi (x)=\min \left(\max \left(\left\{\rho (y):y\in R,y<x\right\}\right),\max \left(\left\{\rho (y):y\in R,y>x\right\}\right)\right) $$

Where *ρ*(*y*) is the read count at genomic coordinate *y*. Bases in the gene region to which the RNA-seq data has been mapped are categorised based on whether they are likely to correspond to exonic or non-exonic regions: a base *x* is considered to be exonic or *on* (i.e. likely included in the mature mRNA) if $\rho (x)>\frac{\chi (x)}{5}$, and intronic or *off* (i.e. likely not included in the mature mRNA) if $\rho (x)<\frac{\chi (x)}{5}$. The coverage score for a gene model *G* = {*G*_1_, …, *G*_*n*_}, where the *G*_*i*_ are alternately exons and introns, is defined as:

$$ C(G)=\frac{1}{n}\ \left(\sum \limits_{G_i\ \mathrm{exonic}}\frac{\ \left|\left\{x\in {G}_i:x\ \mathrm{on}\right\}\right|}{\left|{G}_i\right|}+\sum \limits_{G_j\ \mathrm{intronic}}\frac{\left|\left\{x\in {G}_i:x\ \mathrm{off}\right\}\right|}{\left|{G}_j\right|}\ \right) $$

that is, the average length-adjusted coverage score for each individual feature (exon or intron) in the gene model.

### RNA-seq data

RNA-seq data were downloaded from the Sequence Read Archive, assessed using FastQC [32], trimmed using SeqTK [33] and aligned to the genome with Hi-SAT2 [34, 35] using default parameters and single or paired-end methods as appropriate. Per-base coverage was calculated using SAMtools mpileup [36].

### Subcellular localisation analysis

Subcellular localisation predictions for all datasets were obtained using TargetP [37]. For the plant dataset only, TargetP was run with the –P option to predict chloroplast targeting sequences. The localisation consistency for an orthogroup *O* was calculated as an entropy score across the categories for each gene:

$$ H(O)=-\frac{1}{\left|O\right|}\sum \limits_{C\epsilon \mathcal{C}(O)}\frac{\left|C\right|}{\left|O\right|}\bullet \log \left(\frac{\left|C\right|}{\left|O\right|}\right) $$

where $\mathcal{C}(O)=\left\{{C}_1,\dots, {C}_n\right\}$ is the partition of genes in *O* into their localisation categories.

## Results

### Problem definition, algorithm overview and evaluation criteria

**Fig. 3:**
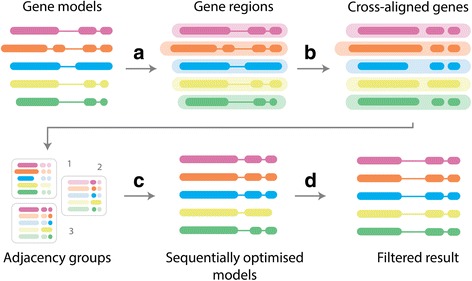
Simplified overview of OMGene workflow. **a**) Gene regions are extracted from around the gene model; **b**) Exonerate is used to cross-align all constituent exons and full open reading frames to construct basic prototype gene models; **c**) The exonic regions from these prototype gene models are sorted into adjacency groups, which are then sequentially optimised using the multipartite choice function; **d**) Results are compared against the original gene models to incorporate potentially overlooked combinations, and filtered under various criteria to produce results

An overview of the OMGene algorithm is provided in Fig. 3. OMGene aims to find the most consistent set of representative gene models for a set of inputted genes by seeking to maximise the agreement of their aligned amino acid sequences, returning the single best gene model for each gene. The algorithm constructs gene models based on relatively simple constraints: AUG for start codons; GU or GC for splice donor sites, AG for splice acceptor sites, and UAA, UGA, or UAG for stop codons. Other features such as codon bias or poly-pyrimidine tracts are not considered. OMGene can also use non-canonical translation initiation and splice sites if inputted by the user as a command-line option.

The input for OMGene is a user-selected set of gene models, in general transfer format (GTF), which are assumed to belong to a single orthogroup. For a given set of species, an orthogroup is the set of genes descended from a single ancestral gene in the last common ancestor of those species [26]: this set may contain paralogous as well as orthologous genes. The suggested pipeline for using OMGene is to determine orthogroups using OrthoFinder [26], and to apply OMGene to each or a chosen subset of these orthogroups.

OMGene uses Exonerate [25] as an initial step to cross-align amino acid sequences from all user-supplied genes to the genomic regions of the genes from all species in question, in order to find conserved translatable features. It then combines this information with the original gene models to produce an initial set of prototype exonic regions, or *gene parts,* for optimisation. The amino acid sequences for these prototype gene models are then aligned, and the constituent gene parts are split into adjacency groups based on overlaps in the alignment (see Implementation). Adjacency groups are sequentially appended to the gene models, and the genetic coordinates are recursively adjusted and assessed to optimise the agreement of the amino acid sequences between species. The resultant gene models are then subject to stringent filtering criteria before the finalised set of gene models are presented as sets of GTF coordinates, amino acid FASTA and coding sequence (CDS) FASTA sequences.

To demonstrate the utility of OMGene, it was applied to orthogroups formed from three sets of test species: a set of five plant species, a set of five mammal species and a set of five fungal species (Table 1). Prokaryotes were not considered, as their protein coding genes lack introns and intergenic regions are either very small or absent and thus gene prediction is comparatively trivial. For plants and fungi, 600 buffer bases either side of each gene model were included, for mammals, which have larger introns, 2000 buffer bases were used. OMGene was applied to orthogroups that contained exactly one gene from each species, referred to as single-copy ubiquitous (SCU) orthogroups. In addition, OMGene was run on the same set but with all genes from three representative species – *A. thaliana*, *H. sapiens* and *S. cerevisiae* – replaced with *de novo* predicted genes, obtained by running the Augustus [24] gene finder on those genomes. These species were chosen as they have the best annotated genomes and thus the existing gene models will provide the best possible training set for Augustus *de novo* prediction. This *de novo* prediction analysis was done to simulate a typical genome-sequencing project where a user has generated a well-trained set of gene models solely using computational prediction.

OMGene was assessed in three ways: RNA-seq data were used to compare the accuracy of gene models before and after application of OMGene, from both coverage (i.e. the proportion of the predicted gene that is encompassed by reads mapped from RNA-seq data) and splice junction perspectives. To assess the accuracy of start codon prediction, OMGene-modified gene models were subject subcellular localisation prediction and the results were evaluated for consistency across the orthogroup. The RNA-seq data used to assess the success of OMGene were downloaded from the NCBI Sequence Read Archive [28] and are listed in Table 2.

### Application of OMGene to publicly available datasets

#### Quantities and nature of changes made

**Fig. 4:**
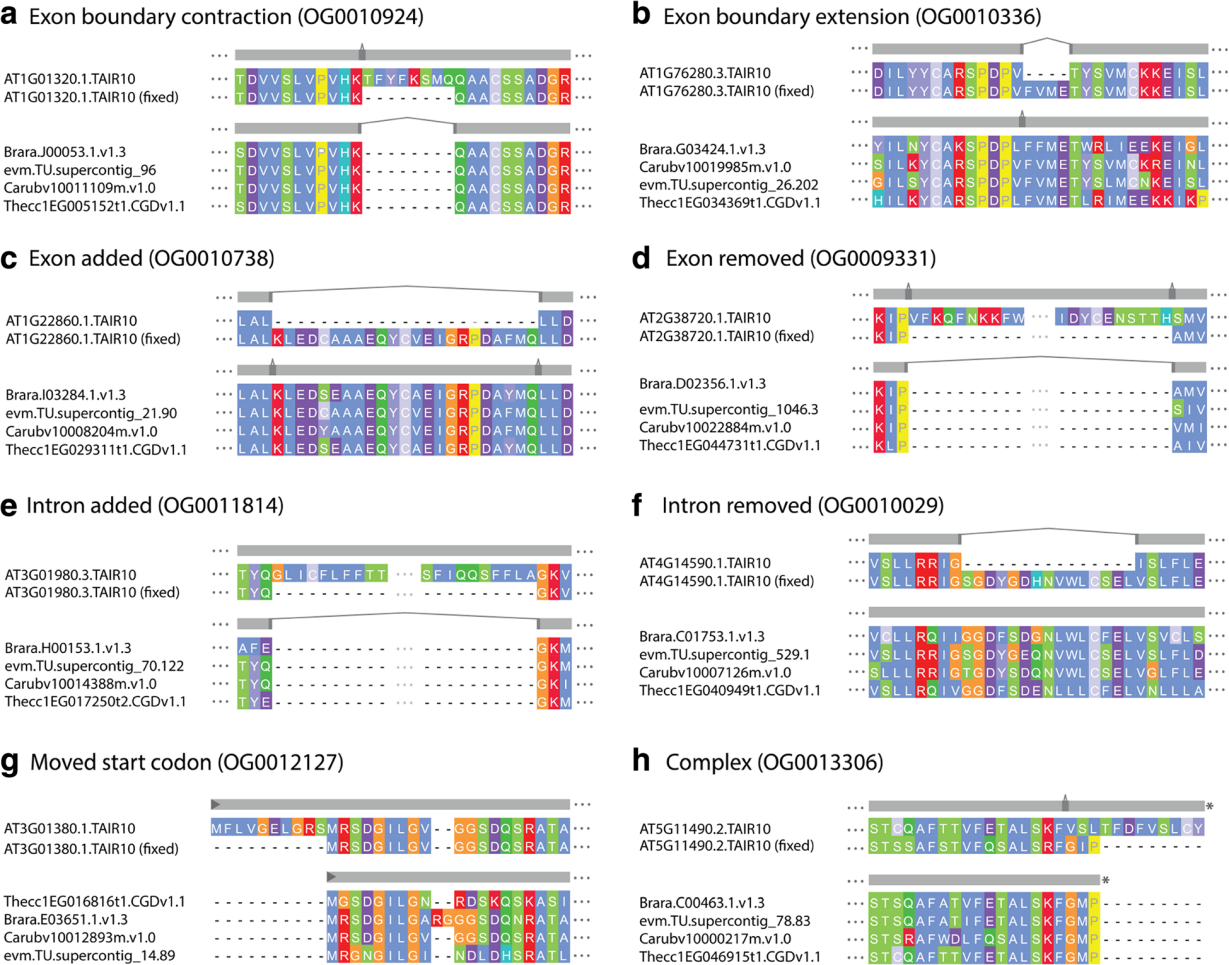
Examples of individual gene model changes for genes in *A. thaliana*. **a**) AT1G01320.1, orthogroup OG0010924, exon extension, splice acceptor side; **b**) AT1G76280.3.TAIR10, orthogroup OG10336, exon contraction, splice acceptor side; **c**) AT1G22860.1, orthogroup OG0010738, novel exon introduced; **d**) AT2G38720.1, orthogroup OG0009331, removed exon; **e**) AT3G01980.3, orthogroup OG0011814, novel intron introduced; **f**) AT4G14590.1, orthogroup OG0010029, intron removed; **g**) AT3G01380.1, orthogroup OG0012127, moved start codon; **h**) AT5G11490.2, orthogroup OG0013306, complex event: exon has been removed and the previous exon boundary has been extended to include the stop codon

**Fig. 5:**
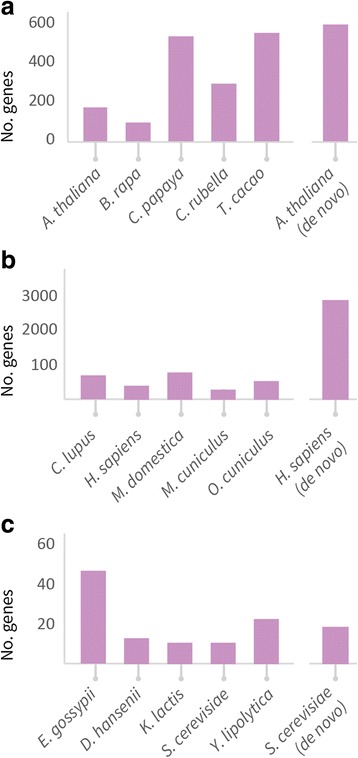
Chart showing the number of changes made. **a**) *C. papaya* and *T. cacao* experienced the most changes in the plant data set. The *de novo* predicted gene models for the *A. thaliana* genome underwent three times more changes than the publicly available one. **b**) The number of changes in mammals was roughly the same as in plants. As expected, *M. musculus* and *H. sapiens* experienced the fewest changes. The *de novo* predicted gene models for *H. sapiens* underwent considerably more changes than the curated genome annotation. **c**) The number of changes made was significantly less for the fungi data set. As for the plants and mammals, the *de novo* predicted genes for *S. cerevisiae* underwent more changes than the curated version

**Fig. 6:**
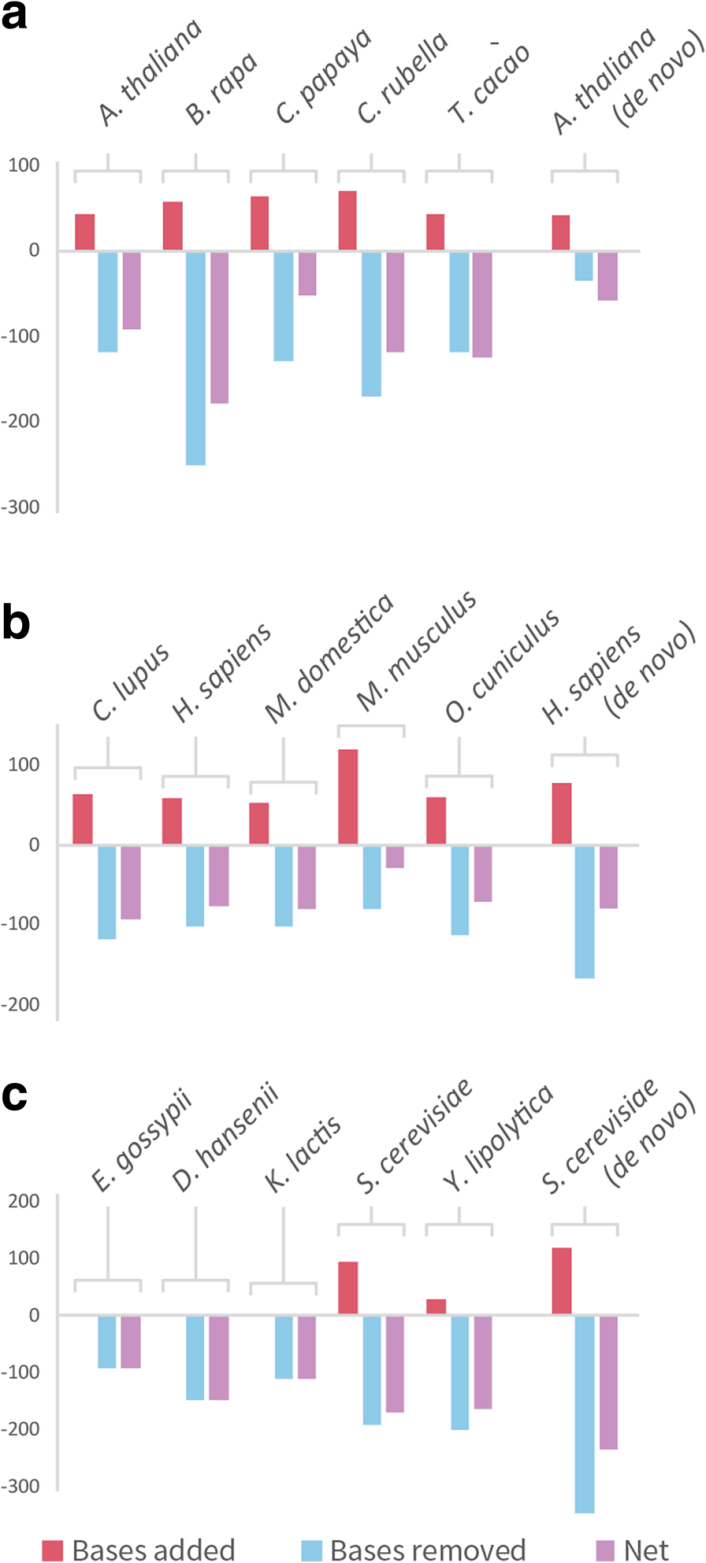
The average number of nucleotides added or removed from gene models as a result of changes made by OMGene. The units of the y-axis are the number of nucleotides. **a**) Average magnitudes of each change for plants; **b**) Average magnitudes for changes made to mammal genes; **c**) Average magnitudes for changes made to fungal genes. In all cases, predicted gene sequences were shortened to a greater extent than they were lengthened.

**Fig. 7:**
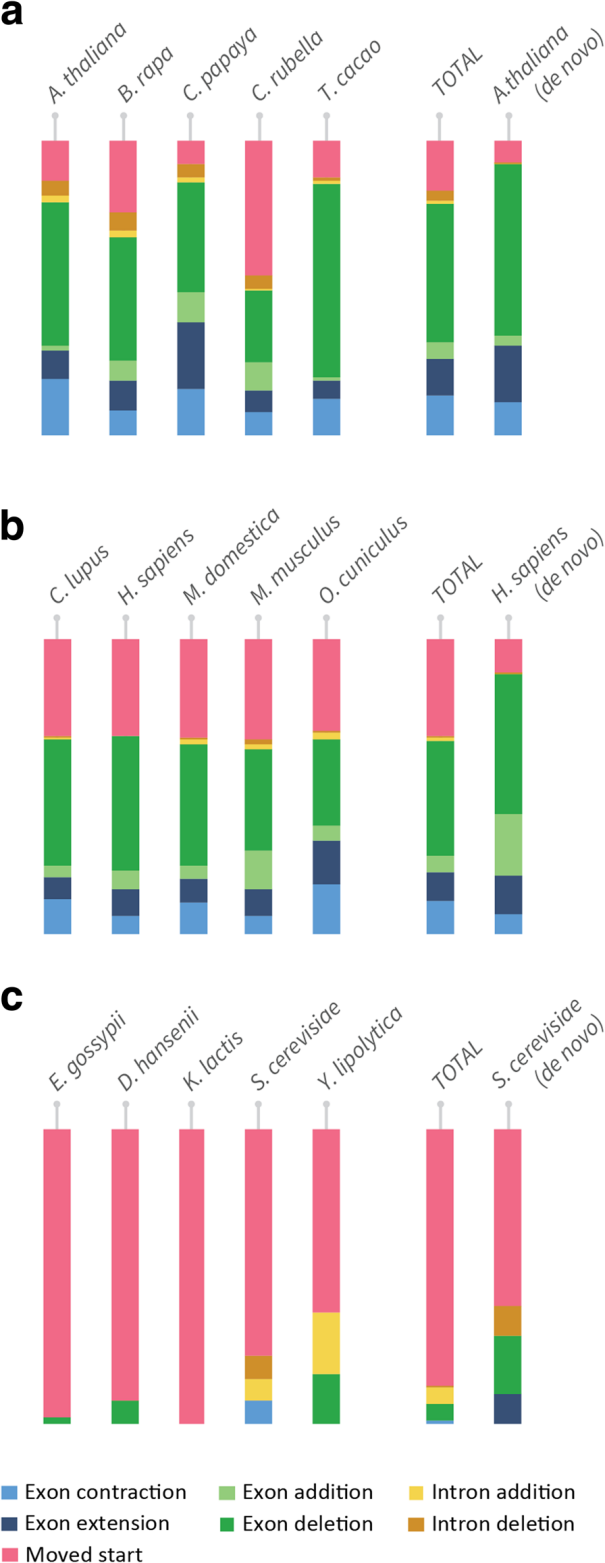
Distribution of types of changes made in the three data sets. **a**) The most common change in plants was exon deletion. **b**) Moved start codons and removed exons were most common in mammals. **c**) In fungi, the most common change was overwhelmingly a moved start codon

The full plant data set contained 3694 SCU orthogroups, containing 18,470 genes. Application of OMGene to this test set resulted in gene model changes to one or more genes in 1543 (41.8%) of these orthogroups. In total, 2017 of the inputted genes (10.9%) were altered. Of these altered versions, 154 genes (7.6% of 2017) were present in the original genome annotations as alternative (non-primary) transcripts for the inputted gene. Figure 4 shows examples of various types of gene model alteration for genes in *A. thaliana*. A full breakdown of per-species change quantities for all species sets can be found in Table 3, Figs. 5 and 6; Table 4 and Fig. 7 show the distribution of the types of changes made. All gene models that were changed by OMGene are included in the supplementary material as a set of GTF files.

**Table 3:** Per-species gene change breakdown

	Species	No. changed genes	Nucleotides added/removed (means per change)			In original annotation as alternative “non-primary” gene model
			+ (mean)	+ (mean)	+ (mean)	
Plants	*A. thaliana*	175	1749 (43)	−23,747 (−118)	−22,139 (−92)	53 (30.3%)
	*B. rapa*	97	1787 (58)	−25,740 (−250)	− 23,953 (− 179)	4 (4.1%)
	*C. papaya*	540	23,820 (65)	−72,053 (− 128)	−48,233 (−52)	0 (0.0%)
	*C. rubella*	298	6568 (71)	−55,005 (− 170)	− 48,437 (−117)	2 (0.7%)
	*T. cacao*	556	3700 (45)	−120,984 (−118)	−117,284 (− 124)	95 (17.1%)
	*TOTAL*	1666	37,624 (61)	− 297,529 (−145)	− 259,905 (−97)	154 (9.2%)
	*A. thaliana* de novo	598	13,623 (42)	−167,038 (−35)	−51,177 (−57)	N/A
Mammals	*C. lupus*	698	8993 (64)	−98,467 (−117)	−89,474 (−91)	375 (53.7%)
	*H. sapiens*	397	4429 (59)	−41,401 (−101)	−36,972 (−76)	218 (54.9%)
	*M. domestica*	787	8637 (53)	−100,256 (−101)	−91,619 (−80)	349 (44.3%)
	*M. musculus*	270	9685 (120)	−19,236 (−79)	− 9551 (−29)	81 (30.0%)
	*O. cuniculus*	534	12,038 (61)	−72,398 (− 112)	−60,360 (−71)	243 (45.5%)
	*TOTAL*	2686	43,782 (67)	−331,758 (− 106)	−287,976 (−76)	1266 (47.1%)
	*H. sapiens* de novo	2907	251,344 (79)	− 952,864 (−167)	− 701,520 (−79)	N/A
Fungi	*E. gossypii*	46	0 (0)	− 4338 (−93)	−4338 (−93)	N/A
	*D. hansenii*	13	0 (0)	− 2080 (−149)	−2080 (−149)	N/A
	*K. lactis*	11	0 (0)	− 1314 (− 110)	−1314 (−110)	N/A
	*S. cerevisiae*	11	93 (93)	− 2483 (− 191)	− 2390 (−170)	N/A
	*Y. lipolytica*	23	117 (29)	− 4186 (− 199)	− 4069 (− 163)	N/A
	TOTAL	104	210 (42)	−14,401 (− 135)	− 14,191 (−127)	N/A
	*S. cerevisiae* de novo	19	601 (120)	− 5561 (− 347)	− 4960 (−236)	N/A

**Table 4:** Summary of gene model change categories

	Species	No. changes	Exon boundary		Exon		Intron		Moved start
			contraction	extension	add	del	add	del	
*Plants*	*A. thaliana*	242	47	23	4	117	5	13	33
	*B. rapa*	134	11	14	9	56	3	8	33
	*C. papaya*	928	148	205	95	345	18	42	74
	*C. rubella*	415	32	32	39	101	1	19	191
	*T. cacao*	949	117	59	9	624	10	13	117
	TOTAL	2668	355	333	156	1243	37	95	448
	*A. thaliana* de novo	1344	151	255	49	780	2	10	97
*Mammals*	*C. lupus*	980	116	72	38	418	9	4	323
	*H. sapiens*	485	31	43	29	223	0	0	159
	*M. domestica*	1148	122	89	56	472	19	3	387
	*M. musculus*	324	20	29	42	111	6	6	110
	*O. cuniculus*	834	139	122	43	245	18	9	258
	TOTAL	3771	428	355	208	1469	52	22	1237
	*H. sapiens* de novo	8883	606	1128	1866	4217	16	17	1033
*Fungi*	*E. gossypii*	46	0	0	0	1	0	0	45
	*D. hansenii*	13	0	0	0	1	0	0	12
	*K. lactis*	11	0	0	0	0	0	0	11
	*S. cerevisiae*	13	1	0	0	0	1	1	10
	*Y. lipolytica*	24	0	0	0	4	5	0	15
	TOTAL	107	1	0	0	6	6	1	93
	*S. cerevisiae* de novo	20	0	2	0	4	0	2	12

The plant species that experienced the highest number of changes were *C. papaya* and *T. cacao*, which is consistent with them being more recently published and less well-studied genomes. For all species, more nucleotides were removed than were added, indicating either that gene models predictions tend to be over-cautious or that OMGene is more proficient at removing material than at adding it in. In terms of the types of changes made, exon deletion was by far the most commonly seen change, followed by moved start codon and exon boundary adjustment (Fig. 7). It should be noted that exon deletion events also encapsulate the separation of erroneously fused gene models, which can contribute many exon deletion events simultaneously.

For the mammal data set, 8771 SCU orthogroups were considered, containing 43,855 genes. Of these, 2100 orthogroups (23.9%) saw some change, with 2686 genes (6.1%) undergoing alterations. Most gene changes occurred in *C. lupus, M. domestica*, and *O. cuniculus*, which, compared to *M. musculus* and *H. sapiens,* are less well studied and more recently annotated. By far the most common alterations here were removed exons and moved start codons, which is likely partially due to the choice of representative gene model for input to OMGene – the longest.

For the full fungal data set, 2710 SCU orthogroups were considered, containing 13,550 genes. Of these, 100 orthogroups (3.7%) exhibited some change, and 109 genes (0.8%) were altered. In this case, the genes in *E. gossypii* were the most commonly altered, consistent again with it being one of the lesser-studied species on the list. By far the most common change type in the fungal data set was a moved start codon, consistent with the fact that splicing is a rare event in fungal genes (on average 5.09 exons for plants, 11.23 exons for mammals, 1.08 exons for fungi).

To simulate a *de novo* genome annotation project, OMGene was also applied to the same three data sets with *de novo* predicted gene models for representative species, *A. thaliana, H. sapiens* and *S. cerevisiae*. These species were chosen as they have the most complete annotations of their respective data sets, and therefore these genes are likely to be the most reliable for training a gene finding algorithm. The genome annotation tool used was Augustus (see Implementation) as it is one of the best and most frequently used gene prediction algorithms.

For the plants data set with Augustus predictions for *A. thaliana*, 3694 SCU orthogroups were considered. Of these, 598 (16.2%) saw some change in an *A. thaliana* gene. Similarly, for the mammal data set, 7311 SCU orthogroups were considered, of which 2907 saw changes in a *H. sapiens* gene. For both *A. thaliana* and *H. sapiens* most changed genes underwent several individual changes. For the fungi data set, 2710 SCU orthogroups were considered. Of these, 19 (0.7%) saw some change in a *S. cerevisiae* gene. Tables 3 and 4 show a full breakdown of the types and amounts of changes made. As expected, in both cases, the total number changes and the average size of change made is greater for the *de novo* predicted gene models than the curated gene models. However, the distribution of types of changes made remained roughly the same.

#### Splice junction and feature coverage analysis

To assess the validity of changes made by OMGene, both the original and the updated gene model sets were compared using publicly available RNA-seq data from the NCBI Sequence Read Archive [28] (see Implementation and Table 2). Data were downloaded for all species except *E. gossypii* and *D. hansenii*, for which no adequate data were available. Each amended gene was assessed in two ways relative to this data: firstly by comparing the exact splice junction locations with RNA-seq derived splice junctions; secondly by evaluating the coverage of exonic regions with RNA-seq. To control for unreliable data, some genic regions were omitted from this analysis. Gene regions in which the RNA-seq data suggested there were indels in the reference genome, or that were within 1000 bp of the end of a contig or scaffold, or that contained 10 or more contiguous “N” nucleotide bases were omitted from the analysis (see Implementation). Regions with these characteristics prevent the creation of reliable gene models, and so are deemed here “unassessable” and not useful for determining gene model accuracy. Gene regions with very low coverage were also omitted from the junction score analysis: gene regions required at least one base with a read depth of 10 or more.

Gene models outputted by OMGene were assessed on whether or not their junction and coverage F-scores (see Implementation) had improved or been reduced. The full results can be seen in Table 5. For the plant data set, OMGene improved the agreement of the gene model with the splice junctions inferred from RNA-seq data for 729 assessable genes, while 125 assessable gene models exhibited reduced agreement (85.3% improved). Similarly, when assessing RNA-seq coverage of gene models OMGene improved the agreement of the models with the data for 1026 genes, while 167 genes exhibited reduced agreement (86.0% improved). For the *de novo* predicted *A. thaliana* genes, the success rates were essentially the same as for the public data (87.3% and 91.1% improved by junction and coverage F-scores respectively), but the absolute quantity of genes exhibiting a changed score increased roughly four-fold. This difference represents the considerable effort and evidence-based curation that has been invested in the *A. thaliana* genome annotation.

**Table 5:** RNA-seq coverage and junction F-scores

	Species	Junction F-score		Coverage F-score	
		Better	Worse	Better	Worse
Plants	*A. thaliana*	94 (87.8%)	13 (12.1%)	109 (91.5%)	10 (8.4%)
	*B. rapa*	24 (63.1%)	14 (36.8%)	29 (56.8%)	22 (43.1%)
	*C. papaya*	246 (82.2%)	53 (17.7%)	344 (83.9%)	66 (16.0%)
	*C. rubella*	90 (89.1%)	11 (10.8%)	186 (91.6%)	17 (8.3%)
	*T. cacao*	275 (88.9%)	34 (11.0%)	358 (87.3%)	52 (12.6%)
	TOTAL	729 (85.3%)	125 (14.6%)	1026 (86.0%)	167 (13.9%)
	*A. thaliana* de novo	422 (87.3%)	61 (12.6%)	475 (91.1%)	46 (8.8%)
Mammals	*C. lupus*	323 (85.9%)	53 (14.1%)	478 (86.28%)	76 (13.72%)
	*H. sapiens*	102 (82.9%)	21 (17.1%)	258 (87.46%)	37 (12.54%)
	*M. domestica*	239 (72.2%)	92 (27.8%)	439 (76.48%)	135 (23.52%)
	*M. musculus*	71 (62.3%)	43 (37.7%)	133 (71.12%)	54 (28.88%)
	*O. cuniculus*	213 (84.2%)	40 (15.8%)	353 (86.10%)	57 (13.90%)
	*TOTAL*	948 (79.2%)	249 (112.5%)	1661 (82.83%)	359 (17.77%)
	*H. sapiens* de novo	832 (89.4%)	99 (10.6%)	2017 (88.74%)	256 (11.26%)
Fungi	*K. lactis*	0 (N/A)	0 (N/A)	9 (100.0%)	0 (0%)
	*S. cerevisiae*	0 (N/A)	0 (N/A)	6 (75.0%)	2 (25.0%)
	*Y. lipolytica*	2 (28.5%)	5 (71.4%)	11 (64.7%)	6 (35.2%)
	TOTAL	3 (37.5%)	5 (62.5%)	30 (75.0%)	10 (25.0%)
	*S. cerevisiae* de novo	4 (100%)	0 (0%)	11 (64.7%)	6 (35.2%)

For the mammals, 948 assessable changed genes (79.2%) had an improved junction score compared to the original gene model. In addition, 82.83% of assessable genes had improved RNA-seq coverage scores. For the *de novo* data, 89.4% of assessable predicted *H. sapiens* had an improved splice junction score after application of OMGene, and 88.74% had an improved coverage score. The numbers of changed genes were again considerably more for the de novo predicted *H. sapiens* data, again indicating the high degree of attention that has been afforded to the annotation of this species.

The results for the fungal data set (see Table 6) were not as good. Notably very few gene models showed any change in junction F-score, with only 8 genes exhibiting a changed score. This is due to the relatively simple exon structure of fungal genes, for which splicing is very rare, and splicing events predicted by OMGene are much less likely to be correct. In this case 3 genes had an improved score, and 5 had a reduced score (37.5% success), with all 5 of the losing genes coming from *Y. lipolytica*. The most common change made to fungal genes was a moved start codon, which, although not detectable in the junction F-score, can be detectable in the coverage F-score. This is reflected in the results, where 30 genes showed an improved coverage F-score and 10 genes showed a worse coverage F-score (75% improved). In the *de novo* case, again the numbers increased while the percentage success remained roughly the same, with 4 (100%) genes improving by junction for *S. cerevisiae* and 11 (64.7%) improving by coverage score. The highly compact nature of fungal genomes, with few exons and limited space between genes means that the accuracy of *de novo* predicted genes is higher than in plants and mammals. Thus the utility of OMGene on these comparatively simpler genomes is limited.

**Table 6:** Subcellular localisation predictions

	Category	No. orthogroups with changed localisation predictions	Entropy score		
			Better	Same	Worse
Plants	Public data	55	42 (76.4%)	5 (7.7%)	8 (14.5%)
	*A. thaliana* de novo	11	9 (81.9%)	0 (0%)	2 (18.2%)
Mammals	Public data	509	444 (87.2%)	19 (3.7%)	46 (9.0%)
	*H. sapiens* de novo	527	458 (86.9%)	23 (4.4%)	46 (8.7%)
Fungi	Public data	7	6 (85.7%)	0 (0%)	1 (14.3%)
	*S. cerevisiae* de novo	1	0 (0%)	0 (0%)	1 (100%)

Many of the cases for which OMGene results differ from RNA-seq evidence are attributable to real biological variability that confounds the evaluation criteria of the algorithm. For example, there are some instances where the most evolutionary conserved splice site was not the splice site observed in the RNA-seq data. Such events, by definition, cannot be detected by OMGene. Furthermore, RNA-seq mapping errors also contributed to reduced scores, as did artefacts resulting from spliced UTRs, and jagged read profiles, particularly in the fungal data, that made some coverage scores difficult to calculate reliably. Finally, the presence of multiple transcript isoforms within the RNA-seq data can reduce the score for a valid transcript even if it is the best choice for that particular gene. While users of OMGene should be aware of these confounding factors, the above data demonstrates that, in general, OMGene is much more likely to improve a given gene model than not even for extensilvey curated genomes.

### Assessment of subcellular localisation predictions for 5′ end analysis

**Fig. 8:**
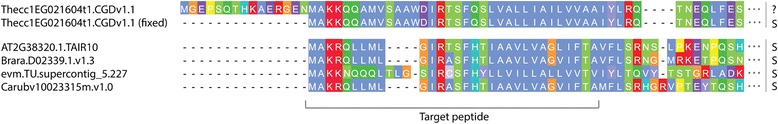
Example change in subcellular localisation prediction for a gene. Thecc1EG021604t1.CGDv1.1 from *T. cacao* has undergone a change in start codon, revealing a signal peptide at its 5′ end. In this case, what was previously assumed to be cytosolic has been found to be targeted to the secretory pathway, the same as the other members of the orthogroup (OG0009265). In this case, the Shannon entropy score for the orthogroup has fallen from 0.72 to 0

Given that genes from the same orthogroup are, by definition, descended from a single ancestral gene, it is reasonable to assume that they should be consistent in their predicted subcellular localisation. Several sub-cellular targeting sequences are located at the N-termini of genes [38], thus one expects genes with inaccurately predicted start codons to yield inaccurate results when assessing their targeting signals. Genes belonging to orthogroups changed by OMGene were assessed to determine whether the changes resulted in increased consistency of the predicted subcellular localisation of all genes in the orthogroup. Targeting predictions were made using TargetP [37], and Shannon entropy was calculated to assess the consistency of the predictions within the orthogroups (see Implementation). Entropy scores were compared only for orthogroups in which at least one gene model was altered by OMGene. An entropy score of 0 indicates that all members of the orthogroup are predicted to localise to the same sub-cellular compartment; the worst possible entropy score given five genes and four possible localisations identified by TargetP (chloroplast, mitochondrion, secreted, cytoplasmic) is $-\frac{2}{5}{\log}_2\left(\frac{1}{5}\right)-\frac{3}{5}{\log}_2\left(\frac{1}{5}\right)\approx 1.92$, indicating that only two of the genes agree. An example orthogroup whose prediction entropy score has been improved by start codon adjustment can be seen in Fig. 8.

The 1543 plant orthogroups in which one or more genes were altered were subjected to subcellular prediction analysis (Table 6). Of these, gene model changes made by OMGene resulted in changes in predicted subcellular localisation for one or more constituent members of 55 orthogroups. In total, 74 improved agreement between gene models (74%), 13 remained the same (13%), and 13% increased disagreement between predicted subcellular localisation of gene models. The effect was more profound for the mammal data: 509 orthogroups showed a change in localisation predictions for one or more of their genes, 444 of which (87.2%) resulted in improved predicted localisation consistency. The data for fungi were sparser: only 7 out of 95 changed orthogroups exhibited a change in subcellular localisation prediction, with 6 of these changes improving the consistency of localisation prediction (85.7%) and 1 increasing disagreement (14.3%).

Similar results were obtained for the *de novo* annotation analysis in plants, although again the data were sparse here. Orthogroups containing the *de novo* predicted *A. thaliana* genes were considered together with the four original genes for the other species. Here, 11 of the *A. thaliana* genes experienced a change in subcellular localisation following application of OMGene. Of the 11 orthogroups containing these, 9 improved consistency (81.9%) and 2 reduced the consistency (18.2%). For de novo *H. sapiens* predictions, 527 of the start codon changes resulted in a change in subcellular localisation prediction change for *H. sapiens*. In 458 (86.9%) of these cases, this improved the consistency of the orthogroup. For the fungal data set, the data were extremely sparse, with only one gene experiencing a change in its targeting prediction, which reduced the consistency for its orthogroup. Thus, although data were sparse for the fungal dataset, in each of the plant, mammal, and fungal datasets the consistency of gene models was on the whole improved from a subcellular targeting perspective.

## Discussion

Here we present OMGene, an automated method for improving the consistency of gene model annotations across species. OMGene is intended for use in computational *de novo* genome annotation projects where no empirical data (such as RNA-seq data) is available to train or correct gene model predictions, or to assist the construction of gene models for genes that are not expressed in the data available. OMGene is also designed to help users who wish to leverage conservation information to correct gene models of a single gene of interest across a set of species. Thus OMGene is suitable for both large and small scale analyses.

OMGene is run as a python script with a tab-delimited input file, each line of which contains the path to a GTF file, containing coordinates for a single gene, and the path to its corresponding genome FASTA file. Full instructions for running OMGene can be found at the OMGene GitHub repository (https://github.com/mpdunne/omgene).

### OMGene results reflect differences in gene model complexity between species sets

To demonstrate the utility and performance characteristics of OMGene, it was applied to three separate datasets of well-annotated plant, mammal and fungal genomes. When applied to the plant data set, OMGene altered the gene models of one or more genes in 41.8% of the orthogroups that were evaluated; when applied to the mammal data set, 23.9% of SCU orthogroups saw some change. In contrast, only 3.7% of orthogroups were subject to modification in the fungal data set. This result reflects the differences in gene model complexity between the three species groups. Specifically, gene models in plants tend to have more exons than fungi (mean = 5.09 exons for plants, 11.23 for mammals, 1.08 exons for fungi) and thus there is considerably more potential for gene model variation in plants and mammals than in fungi. In light of this it was unsurprising that the most frequently observed change made in fungi was a change in choice of start codon. This is also reflected in the high number of removed exons from plant and mammal genes, which is contributed to partly by the separation of erroneously fused adjacent genes.

### OMGene works well on complex gene models

The changes made by OMGene were assessed relative to splice-mapped RNA-seq data to assess the level to which it had improved the gene models. For the plant data set, the results from OMGene clearly resembled the empirical data more closely, with 85.4% and 86.0% of genes improving in terms of their splice junctions and their coverage respectively. The profiles were different for different species, with many more changes being made for *C. papaya* and *T.cacao;* in addition the number of successes for *B. rapa* was slightly lower than for the other species.

In mammals, the three non-model species, *C. lupus*, *M. domestica*, and *O. cuniculus* faired well in terms of their junction scores. *H. sapiens* genes also typically scored highly, but *M. musculus* genes did not do so well. The reason why mouse genes changes were less successful than human gene changes is unknown. These two species also saw considerably fewer gene model changes than the other species. In all cases, mammal genes were mostly contracted via the removal of exons or by moving the start codon – this is at least partly due to biases in the choice of longest gene model as representatives for these genes.

The number of junction changes made for the fungal data set was considerably lower: only 8 changed genes had an altered junction F-score, 62.5% of which become worse after OMGene. Though this is less than the plant data set, it should be noted that the resolution of this data set does not lend itself to accurate conclusions about the general validity of changes made to fungal genes. The resolution and success rate for fungal genes from a coverage perspective was slightly higher, with 75% of the genes with changed scores improving. The low resolution of junction data for fungal genes reflects the rarity of complex gene models in these species, and thus the low likelihood that deviations from simple, single-exon gene models are correct. Thus, while OMGene does not always produce gene models that agree with transcriptome data, it does improve the overall quality of gene model annotations even for relatively simple fungal genomes.

The improvements in gene model accuracy made by OMGene for the *de novo* predicted gene models were much the same as for the publicly available, curated gene models. However, the number of changes made to the *de novo* predicted set was much greater, indicating that the considerable labour that has been applied to these model organisms has successfully controlled for potential errors. It should be noted that, although OMGene managed to improve many of the gene models outputted by Augustus, the two agreed in most cases (86.1%, 60.3% and 98.6% for plants, mammals and fungi respectively), indicating that the basic implementation of a well-trained Augustus *de novo* prediction produces genes that are highly consistent between species.

### OMGene improves the consistency of subcellular localisation predictions

In addition to assessment of coverage and splice junctions, gene models were assessed by the consistency of their predicted subcellular localisation. Given that the orthogroups used in this analysis comprise ubiquitously conserved single copy genes, it is logical to assume that these genes should generally have the same subcellular localisation. For the full plant data set, of all orthogroups whose genes had different subcellular targeting predictions after application of OMGene, 76.4% had improved intra-orthogroup consistency, with 85.5% either improving or remaining the same. In mammals, 87.2% of orthogroups whose subcellular localisation predictions were changed by OMGene showed improved consistency. For the full fungal data set, although the data were sparse, 85.7% of the orthogroups considered had improved consistency.

The results for the plant data set were similar for the *de novo* annotated set (85.7% improvement), as was also the case for mammals (86.9% improvement). For fungal orthogroups containing *de novo* predicted *S. cerevisiae* genes, the only gene whose localisation prediction changed caused the consistency of its orthogroup to decrease, however the resolution of the data in this case is not sufficient to draw any conclusions. Thus, application of OMGene improves the accuracy of start codon specification in *de novo* predicted gene models.

## Conclusions

When applied to publicly available plant, mammal and fungal data sets, OMGene demonstrates proficiency in improving gene models from multiple perspectives. Due to stringent filtering criteria, it does not fix all errant gene models, however the gene models that it does fix represent an improvement the majority of the time. The overall improvement is larger for genomes with complex gene models.

## Availability and requirements

**Project name**: OMGene.

**Project home page**: https://github.com/mpdunne/omgene

**Operating system**: Unix.

**Programming languages**: Python v2.7.x.

**Other requirements**: Bedtools, Exonerate, Python packages (scipy, numpy, pyfaidx, networkx, BioPython).

**License**: GPLv3.

## Abbreviations

CDS: Coding sequence
GTF: General transfer format
SCU: Single copy, ubiquitous
